# Enhancement of Glucosinolate Formation in Broccoli Sprouts by Hydrogen Peroxide Treatment

**DOI:** 10.3390/foods11050655

**Published:** 2022-02-23

**Authors:** Adriana Vanegas Torres, Nimrod Tish, Victor Rodov

**Affiliations:** 1 Department of Postharvest Science, Agricultural Research Organization (ARO)—The Volcani Institute, Rishon LeZion 7505101, Israel; adriana.vanegas@mail.huji.ac.il (A.V.T.); nimrod.tish@mail.huji.ac.il (N.T.); 2 The Robert H. Smith Faculty of Agriculture, Food and Environment, The Hebrew University of Jerusalem, Rehovot 7610001, Israel; 3 The Goodman Faculty of Life Sciences, Bar-Ilan University, Ramat Gan 5290002, Israel

**Keywords:** *Brassica oleracea* var. *italica*, sprouts, elicitation, phytonutrients, glucosinolates, phenolic compounds, wild rocket, *Diplotaxis tenuifolia*, gene expression, sulfur metabolism

## Abstract

Broccoli sprouts are known as a rich source of health-beneficial phytonutrients: glucosinolates and phenolic compounds. The production of phytonutrients can be stimulated by elicitors that activate the plant stress response. The aim of this study was enhancing the nutritional value of broccoli sprouts using hydrogen peroxide (H_2_O_2_) as an elicitor. Daily spraying with H_2_O_2_ (500–1000 mM) enhanced the accumulation of glucosinolates, doubling their content in the cotyledons of 16/8 h photoperiod-grown 7-day sprouts compared to the water-treated controls. The application of H_2_O_2_ on dark-grown sprouts showed a smaller extent of glucosinolate stimulation than with light exposure. The treatment affected sprout morphology without reducing their yield. The H_2_O_2_-treated sprouts had shorter hypocotyls and roots, negative root tropism and enhanced root branching. The activated glucosinolate production became evident 24 h after the first H_2_O_2_ application and continued steadily until harvest. Applying the same treatment to greenhouse-grown wild rocket plants caused scattered leaf bleaching, a certain increase in glucosinolates but decline in phenolics content. The H_2_O_2_ treatment of broccoli sprouts caused a 3.5-fold upregulation of APK1, a gene related to sulfur mobilization for glucosinolate synthesis. Comparing the APK1 expression with the competing gene GSH1 using sulfur for antioxidant glutathione production indicated that glutathione synthesis prevailed in the sprouts over the formation of glucosinolates.

## 1. Introduction

Plants from the *Brassicaceae* family such as broccoli, cabbage, Brussels sprouts, kale, mustard, radish, etc., are valuable sources of health-beneficial natural compounds for human nutrition (phytonutrients), helpful for preventing chronic diseases [1,2,3,4]. The *Brassicaceae* phytonutrients include, in particular, phenolic compounds known as potent antioxidants [5] and glucosinolates, a group of nitrogen- and sulfur-containing anti-cancer secondary metabolites [6]. Glucosinolates are found in plant vacuoles and comprise a β-D-thioglucose group, a sulfonated oxime group and a side chain derived from an amino acid [7]. Depending on the amino acid in the side chain, the glucosinolates can be classified into aliphatic (based on methionine), indolic (based on tryptophan) and aromatic (based on tyrosine or phenylalanine) glucosinolates [8].

Glucosinolates contribute to the health benefit of the *Brassicaceae* crops through their hydrolysis by an enzyme myrosinase that generates bioactive volatile pungent compounds isothiocyanates along with other products, e.g., nitriles [9,10]. The hydrolysis is activated upon tissue disruption by chewing, cutting, attack of phytopathogens, herbivores or gastrointestinal microbiota [11,12,13]. One prominent glucosinolate is glucoraphanin, a precursor of an isothiocyanate sulforaphane possessing potent anti-cancer activity [14]. Young sprouts of broccoli were found to be an exceptionally rich source of glucosinolates and isothiocyanates containing 10–100 times higher levels of glucoraphanin than the mature plants [15]. Broccoli sprouts were shown to possess high antioxidant, anti-proliferative and antibacterial properties [16]. In general, edible sprouts are known as functional foods rich in phytonutrients [17,18].

A number of genes are involved in glucosinolate formation [19], in particular MYB transcription factors regulating the biosynthesis of aliphatic and indole glucosinolates [20,21,22]. Other relevant genes are from the Cytochrome P450s family (CYPs) involved in the core glucosinolate biosynthesis, e.g., CYP79F1 for aliphatic path and CYP79B3 for indole path [20]. Duarte-Sierra et al. [23] showed the upregulation of CYP79 family genes after oxidative stresses in broccoli florets. Guo et al. [24] revealed that heat and hypoxia stresses in broccoli sprouts resulted in the enhanced accumulation of aliphatic glucosinolates and expression of relevant genes including CYP83A1. APS kinases (APKs) are another group of redox-regulated genes mobilizing sulfur for glucosinolate synthesis [25]. Alternatively, sulfur may be used for the production of antioxidant glutathione (GSH) involved in the detoxification of reactive oxygen species [26].

The biological role of sulfur-containing and phenolic phytonutrients in *Brassicaceae* plants is associated with defense against biotic (pathogens, pests, herbivores) and abiotic (cold, heat, irradiation) stresses [12]. Therefore, the content of phytonutrients can be enhanced by treatment with elicitors, i.e., factors that induce plant defense responses through various signaling pathways [27,28]. In particular, the defense responses can be elicited by interventions associated with oxidative stress, e.g., hydrogen peroxide (H_2_O_2_) or ultraviolet light [29,30]. Reactive oxygen species (ROS) such as H_2_O_2_ are naturally produced by cells in course of reduction/oxidation (redox) reactions, and their levels are controlled by antioxidant enzymes [31]. H_2_O_2_ is involved in many physiological processes such as seed germination, acting both as a plant development regulator and a direct antimicrobial protection agent [32,33]. At the same time, H_2_O_2_ acts as a signaling molecule inducing the plant’s defense response towards biotic and abiotic stresses [34]. Depending on the concentration used, exogenous H_2_O_2_ can induce a signaling cascade leading to defense molecules (e.g., phytoalexins) production or cause phytotoxic damage [35]. It was shown that the application of H_2_O_2_ increased the content of polyphenols in lentil and mungbean sprouts [29,30]. However, no data are available about the effect of H_2_O_2_ on glucosinolates content in *Brassicaceae* sprouts. The only somewhat relevant information is the conference report of Arriaga-Madrid et al. [36] about the increased accumulation of glucosinolates in some organs (primarily flowers and seeds) of adult mustard plants sprayed with H_2_O_2_ throughout the greenhouse crop cycle.

In this work, we investigated the possibility of improving the nutritional value of broccoli sprouts by the application of hydrogen peroxide as elicitor enhancing the production of phytonutrients—glucosinolates and phenolic compounds. In addition, the performance of elicitor treatment that showed the highest efficacy with broccoli sprouts was examined with greenhouse-grown wild rocket plants.

## 2. Materials and Methods

### 2.1. Materials

Organic broccoli seeds (*Brassica oleracea* cv. Calabrese) were purchased from Eco-Store, Tel Aviv-Yafo, Israel. Hydrogen peroxide, palladium (II) chloride (PdCl_2_), Folin-Ciocalteu’s phenol reagent, sinigrin and gallic acid standards and Spectrum™ Plant Total RNA Kit were purchased from Sigma Israel, Rehovot, Israel. Maxima first strand cDNA synthesis Kit with dsDNAase and Fast SYBR green Master mix were purchased from Thermo Fisher Scientific.

### 2.2. Plant Material and Sprouting Procedure

**Figure 1:**
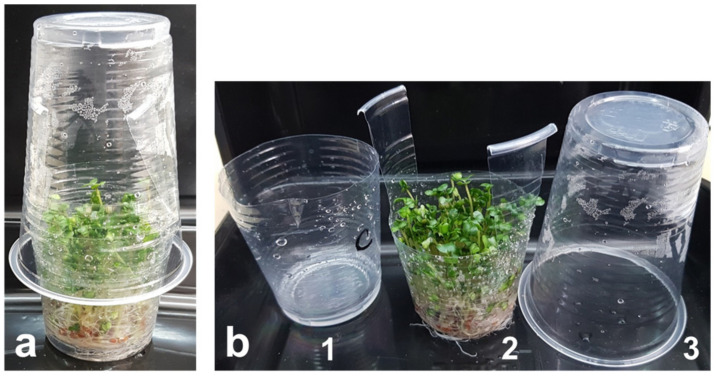
Sprouting container: assembled (**a**) and disassembled (**b**) into three parts: 1—non-perforated outer drainage cup; 2—perforated inner growing cup; and 3—transparent cover. During the first three days of sprouting, a light-impermeable cup completely covering the container was used instead of the transparent cover.

Broccoli seeds were sterilized in 2% of calcium hypochlorite as recommended by FDA [37], rinsed 7–8 times with distilled water and soaked for 4 h. The seeds were germinated and further grown in custom-made two-layer containers comprising inner perforated and outer non-perforated plastic cups (Figure 1), approximately 300 seeds per container. The seeds were spread on the bottom of the perforated cup inserted in the non-perforated one that contained ca. 1 mL water to maintain a moist environment without covering the seeds.

The sprouting containers were kept for 7 days at 22 °C in an illuminated chamber with 16/8 h light–dark cycle with a photosynthetically activity radiation (PAR) level of 150 µmol m^−2^ s^−1^. During the first three days, the containers were covered with light-impermeable paperboard cups to avoid light exposure. Starting from the morning of day 3, the paperboard covers were changed for transparent plastic cups to allow light exposure of the sprouts. Once a day, the sprouts were rinsed with distilled water and sprayed (ca. 1.5 mL per container) with water or H_2_O_2_. In the control, the sprouts were sprayed with distilled water throughout the growing cycle. In the treatments, the germinating seeds were sprayed with water on the first day and from day 2 until day 6 with H_2_O_2_ (20 to 1000 mM in different treatments). The H_2_O_2_ solutions were freshly prepared from recently purchased commercial 30% hydrogen peroxide whose concentration was verified spectrophotometrically at a wavelength of 240 nm and extinction coefficient of 43.6 M^−1^ cm^−1^ [38].

Modified procedures of sprout growing and treatment were tested in some trials. One modification included a single H_2_O_2_ application on day 2 followed by water sprays until day 6. In another case, the sprouts were repeatedly sprayed with H_2_O_2_ as described above, but they were consistently kept under light-impermeable covers until day 7 without light exposure, except for brief spraying sessions once a day. Irrespectively of the growing procedure peculiarities, the seven-day-old sprouts were harvested, weighed and lyophilized to obtain the dry material. The lyophilized material was weighed again, ground in a mortar to obtain a dry powder that was put in Eppendorf tubes and quickly frozen in liquid nitrogen. If the powder was not used the same day, it was stored at −80 °C to avoid myrosinase activity.

In the trials with wild rocket (*Diplotaxis tenuifolia*) plants, the rocket seeds were sown in a planting mix. During the first 2 days, they were kept in the dark at 23 °C. Then, the seedlings were exposed to nature light conditions for about 14 h. The plants were watered every day. At 12 days of age, the seedlings were transplanted to pots containing the planting mix. The pots belonging to the control and treatment groups were kept in separate bins. Seven days after the transplantation, when the rosette leaves were well-developed, the treatment with 1000 mM of H_2_O_2_ started. The control group was sprayed with distilled water. The H_2_O_2_ or water sprays were applied daily for 4 days, and the following day after the last treatment, the leaves were harvested, weighed, promptly frozen in liquid nitrogen and lyophilized. The dried material was weighed, put in microfuge tubes with addition of grinder beads and crushed using a Geno/Grinder 2666. The dry powder was stored at −80 °C until the analysis.

### 2.3. Extraction of Phytochemicals

The procedure of Villarreal-García et al. [39] was followed for extracting the glucosinolates and phenolic compounds from the dry powder. Aqueous 70% methanol (10 mL) heated to 70 °C was added to 0.2 g of the freeze-dried powder. Taking care of the myrosinase deactivation, the samples were incubated for 30 min at 70 °C with vortexing every 10 min. After cooling down to room temperature, the samples were centrifuged (13,000× *g*, 10 min, 4 °C), and the supernatant collected for the analysis of glucosinolates and phenolic compounds [40].

### 2.4. Total Glucosinolates Analysis

Total glucosinolates (GSL) content was determined via palladium colorimetric analysis. Following Ishida et al.’s [41] procedure, the samples were prepared by mixing 0.2 mL of the crude extract with 0.3 mL of distilled water and 3 mL of 2 mM PdCl_2_ (35.5 mg PdCl_2_ + 168 µL of hydrochloric acid + 100 mL of distilled water). After the reagent addition and mixing, the samples were incubated at room temperature for 1 h, and absorbance at 425 nm was measured with a spectrophotometer Genesys 10S UV-VIS (Thermo Scientific, Waltham, MA, USA). A blank sample comprised distilled water and PdCl_2_ solution. The calculation of total GSL content (µmol g^−1^) was based on the absorbance difference between the sample and the blank using the calibration curve prepared with sinigrin as a standard glucosinolate.

### 2.5. Total Phenolic Compounds Analysis

Phenolic compounds were analyzed by the Folin–Ciocalteu method [42]. The samples were prepared by mixing 0.1 mL of the crude extract with 6 mL of distilled water and 0.5 mL of FC reagent. After incubation for 3 min, 1.5 mL of sodium carbonate Na_2_CO_3_ solution (20%) was added, mixed again and filled with water to obtain 10 mL of volume. The samples were incubated at room temperature for 2 h, and absorbance at 765 nm was measured with a spectrophotometer Genesys 10S UV-VIS. A blank sample was prepared with the same procedure without the addition of the crude extract. The calculation of total phenolic compounds content (mg g^−1^) was based on absorbance difference between the sample and the blank using the calibration curve prepared with gallic acid as a standard phenolic following the procedure of Slinkard & Singleton [43]. Total phenolic content was expressed as mg gallic acid equivalents (GAE) per g sprouts mass (dry or fresh).

### 2.6. RNA Extraction and Real Time-PCR (RT-qPCR) Analysis

RNA was extracted from sprouts after a single treatment with 1000 mM H_2_O_2_ or water (control) applied at day 2 with subsequent incubation of 15 h in the dark at 22 °C. The sprouts were harvested and immediately frozen in liquid nitrogen to avoid RNA degradation. Then, samples were lyophilized and ground using a mortar and pestle. The crushing was performed using liquid nitrogen, preventing the samples from thawing out. The sprout powder was stored at −80 °C. RNA extraction was performed with 10 mg of powder and following the protocol of Spectrum™ Plant Total RNA Kit. The RNA yield was verified by NanoDrop ND-1000 spectrophotometer (NanoDrop Technologies, Wilmington, DE, USA), and electrophoresis was run in order to verify the quality of the RNA. cDNA was synthesized using Maxima first strand cDNA synthesis Kit with dsDNAase. For amplification of the cDNA, primers were designed using Integrated DNA Technologies (IDT) and Primer3Plus software according to the gene sequence of *Brassica oleracea* var. *italica*, *Brassica oleracea* var. *oleraceae* and *Arabidopsis thaliana*. Additionlly, primers already evaluated were selected, such as the housekeeping gene β-actin [24] and MYB29 [22]. In order to run the RT-PCR, 10 µL of mixture was prepared for each well on a MicroAmp Fast Optical 96-Well Reaction Plate, including 5 µL of Fast SYBR Green Master mix, 2.2 µL RNAse-free water, 0.3 µL primer mix (forward and reverse primers) and 2.5 µL of diluted cDNA. RT-PCR was run on StepOne Plus Real-Time PCR system and analyzing using StepOne software v. 2.2.2.

### 2.7. Statistical Analysis

The trials and assays were carried out in triplicates. Trials were repeated at least twice with similar outcomes, and the results of typical trials are presented. The data were evaluated by analyzing variance (ANOVA) using JMP Pro 2015 software (SAS Institute, Cary, NC, USA). In addition, a Tukey’s HSD test was used for a post hoc pairwise comparison. In some analyses, such as RT-qPCR, Student’s *t*-test was run. Data were expressed as means ± standard error, and *p* value of <0.05 was considered significant.

## 3. Results

### 3.1. Effects of Hydrogen Peroxide (H_2_O_2_) on Broccoli Sprouts

Hydrogen peroxide was initially tested in four concentrations (20, 50, 100, 200 mM). None of these H_2_O_2_ concentrations showed a statistically significant effect on the sprouts’ yields. The total contents of glucosinolates and of phenolic compounds tended to decrease when the sprouts were exposed to the lowest H_2_O_2_ dose (20 mM) and to return to the control level with higher concentrations. Furthermore, at 100 and 200 mM H_2_O_2_, the glucosinolate content values were ca. 20% higher than in the control, although the difference was not statistically significant (data not shown).

**Figure 2:**
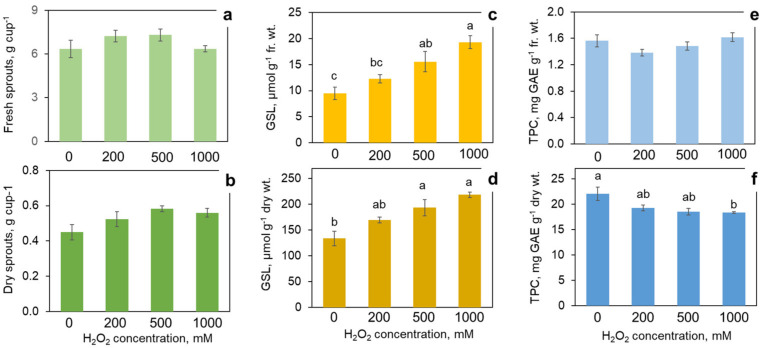
Effect of hydrogen peroxide H_2_O_2_ treatment in concentrations of 0 (control), 200, 500 and 1000 mM on the yield of fresh (**a**) and dry (**b**) 7-day broccoli sprouts and on the content of phytonutrients: glucosinolates (GSL) on fresh (**c**) and dry (**d**) weight basis, and total phenolic compounds (TPC) on fresh (**e**) and dry (**f**) weight basis. Error bars represent standard errors of three independent biological replications. Bars marked by different letters indicate significantly different values according to post-hoc Tukey’s HSD test (*p* ≤ 0.05). Absence of letters means that the values were not significantly different according to ANOVA test.

Taking into account that the first H_2_O_2_ application showed a certain increase in glucosinolates with the highest H_2_O_2_ concentration used (200 mM), even higher H_2_O_2_ doses were tested in the new trial, bringing a significant enhancement of the glucosinolate accumulation. H_2_O_2_ at 500 and 1000 mM increased the glucosinolate content by 64% and 104%, respectively, on a fresh weight basis, or 45% and 63%, respectively on a dry weight basis (Figure 2c,d). With 200 mM H_2_O_2_, the increase was not statistically significant, similar to the results of the previous trial. The phenolics content calculated on a fresh weight basis did not significantly differ at any concentration of H_2_O_2_. However, on the dry weight basis, the highest H_2_O_2_ concentration (1000 mM) caused a small but significant reduction of 17% in the phenolics content as compared to the control (Figure 2e,f).

**Figure 3:**
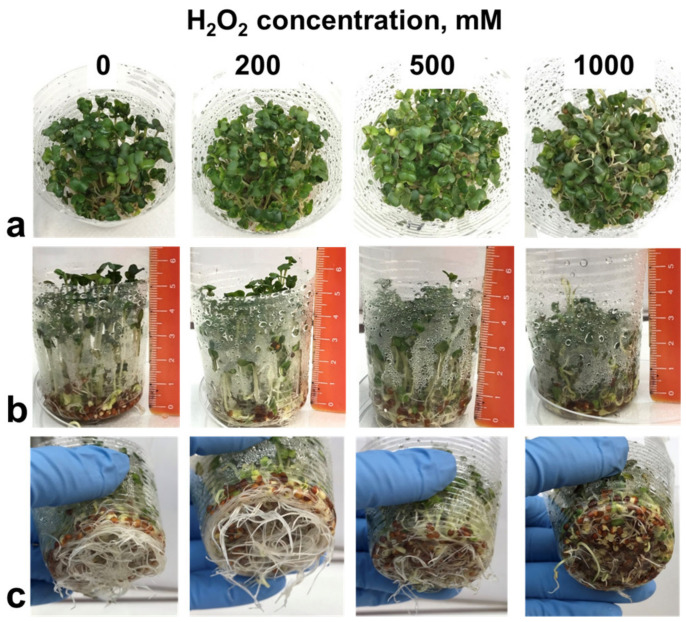
Effect of hydrogen peroxide H_2_O_2_ treatment in concentrations of 0 (control), 200, 500 and 1000 mM on the appearance of 7-day broccoli sprouts: view from the top (**a**), side (**b**) and bottom (**c**). Note the negative root tropism in the 1000 mM H_2_O_2_ treatment.

**Figure 4:**
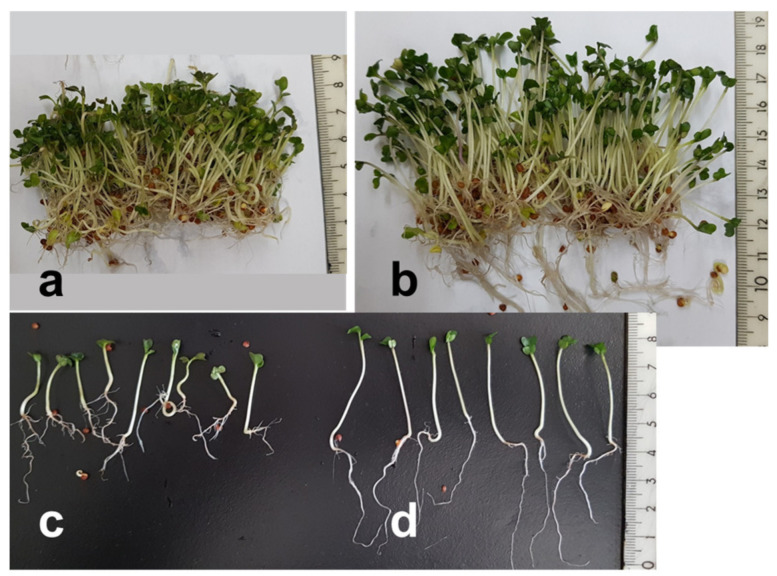
Effect of hydrogen peroxide treatment on the appearance of 7-day broccoli sprouts. Content of one growing container: treated with 1000 mM H_2_O_2_ (**a**) and control (**b**); individual sprouts: treated with 1000 mM H_2_O_2_ (**c**) and control (**d**).

The high concentrations of H_2_O_2_ caused morphological changes in the broccoli sprouts most evident with 1000 mM H_2_O_2_ (Figure 3). The sprouts treated with 1000 mM of H_2_O_2_ were noticeably shorter than the control. The treatment resulted in a marked inhibition of root growth. Remarkably, the roots demonstrated negative tropism when some of them grew upwards, as if they were trying to escape the high concentration of H_2_O_2_ accumulated on the bottom of the cup after spraying. In contrast with the inhibited primary root elongation, there was a prominent stimulation of lateral root development (Figure 4). In spite of the obvious effect of the high H_2_O_2_ concentrations on the sprout morphology, they did not cause a significant change in the fresh and dry weight yield (Figure 2a,b). It can be inferred that the treatment resulted in thicker and/or denser sprouts (Figure 4).

### 3.2. Factors Affecting the Glucosinolate Accumulation in H_2_O_2_-Treated Sprouts

**Figure 5:**
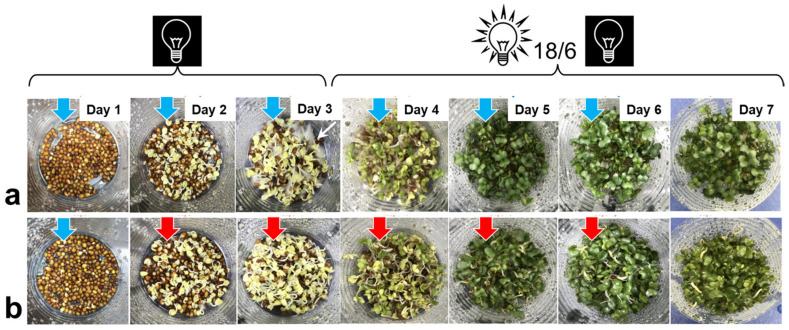
Appearance evolution of broccoli sprouts treated with water (control, (**a**)) or 1000 mM H_2_O_2_ (**b**) during the 7-day growing cycle at 22 °C. Water spraying is marked by blue arrows and H_2_O_2_ spraying by red arrows. Until day 3, the sprouts were incubated in the dark and afterwards under a 18/6 h photoperiod. Note fluffy root hairs development in the control on day 3 (marked by white arrow) and negative root tropism in H_2_O_2_-treated sprouts starting from day 4.

A separate trial investigated the time course of sprout development and glucosinolate accumulation in H_2_O_2_-treated vs. control samples during the 7-day growth cycle. The appearance evolution of the plant material during the growth cycle is presented in Figure 5. One characteristic morphological feature of the control sprouts was the development of fluffy root hairs on day 3 that gave more surface to absorb water. However, the root hairs were much less visible in the H_2_O_2_-treated sprouts (Figure 5). Another morphological peculiarity of the H_2_O_2_-treated sprouts, the negative root tropism, became evident on day 4. Figure 3, Figure 4 and Figure 5 show that spraying with 1000 mM H_2_O_2_ caused no visible phytotoxic effects (e.g., bleaching) on broccoli cotyledons.

**Figure 6:**
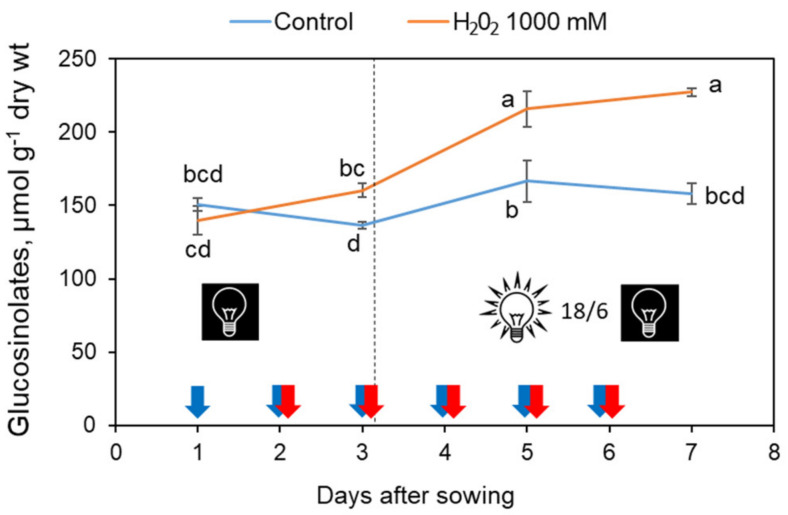
Time course of total glucosinolate content (on a dry weight basis) in broccoli sprouts sprayed with water (control) or 1000 mM H_2_O_2_ during the 7-day growing cycle at 22 °C. Water spraying is marked by blue arrows and H_2_O_2_ spraying by red arrows. On day 1, all germinating seeds were sprayed with water. Starting from day 2 until day 6, the control sprouts were sprayed with water and the experimental sprouts with H_2_O_2_. Until day 3, the sprouts were incubated in the dark and afterwards under a 18/6 h photoperiod. Different letters indicate significantly different values according to post-hoc Tukey’s HSD test (*p* ≤ 0.05).

The time course of glucosinolates content on a dry weight basis is presented in Figure 6. The sample taken for the analysis on day 1 (i.e., one day after sowing) comprised germinating and non-germinating seeds, including the seed coats, while at further sampling points (days 3, 5 and 7), only sprouts separated from seed coats and from non-germinated seeds were analyzed. In the control, the glucosinolate content on a dry weight basis stayed constant throughout the trial period and did not exceed the initial amount found in the seeds on day 1 (Figure 6). On the contrary, just one day after the first H_2_O_2_ spray, the glucosinolate level in the treated sprouts was significantly higher than in the control. Stimulated by repeated H_2_O_2_ exposures, the glucosinolate content continued to increase reaching at the end of the trial a ca. 50% higher value than in the control. The fastest glucosinolate accumulation was evident after transferring the sprouts to light (between days 3 to 5).

**Figure 7:**
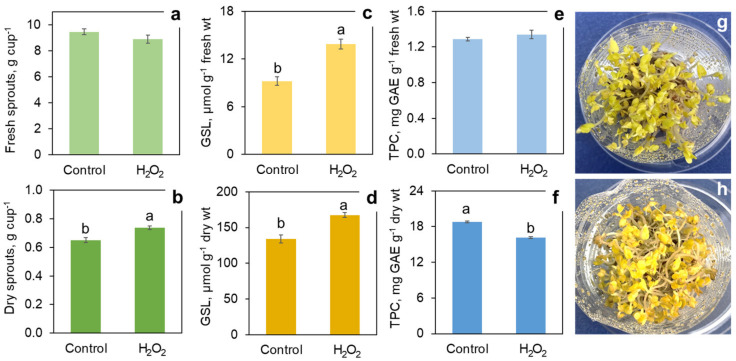
Effect of hydrogen peroxide treatment (1000 mM H_2_O_2_) in comparison with water spray (control) on yield, phytonutrients content and appearance of 7-day broccoli sprouts constantly grown in the dark at 22 °C. Yield: fresh sprouts (**a**), dry sprouts (**b**); total glucosinolates (GSL) content on fresh (**c**) and dry (**d**) weight basis; total phenolic compounds (TPC) content on fresh (**e**) and dry (**f**) weight basis. Sprout appearance: control (**g**) and H_2_O_2_ treatment (**h**). Error bars represent standard errors of three independent biological replications. Bars marked by different letters indicate significantly different values according to post-hoc Tukey’s HSD test (*p* ≤ 0.05). Absence of letters means that the values were not significantly different according to ANOVA test.

The single application of H_2_O_2_ on day 2 resulted in certain changes of hypocotyl and root morphology, but they were not as drastic as in the case of repeated daily spraying. The sprouts that underwent a single H_2_O_2_ treatment showed at the end of the 7-day growth cycle 11–19% higher glucosinolate accumulation values, but this increase was not statistically significant (data not shown). On the other hand, the repeated application of H_2_O_2_ on etiolated sprouts continuously kept in the dark showed a significant stimulation of glucosinolate accumulation but still to a smaller extent than with light exposure by 50% and 25% on a fresh and dry weight basis, respectively (Figure 7). At the same time, the H_2_O_2_ treatment resulted in a significant 14% decrease in the content of phenolic compounds on a dry weight basis. The etiolated sprouts were longer than the light-exposed ones and yellow in color but followed a similar morphological pattern in response to H_2_O_2_: relatively shorter hypocotyls and roots, enhanced root branching and negative tropism. In addition, the treated sprouts had a somewhat darker yellow color, with burning signs on cotyledon edges (Figure 7). In spite of their shorter hypocotyls, the H_2_O_2_-treated etiolated sprouts showed a significant 13% increase in dry weight yield compared to the controls (Figure 7).

**Figure 8:**
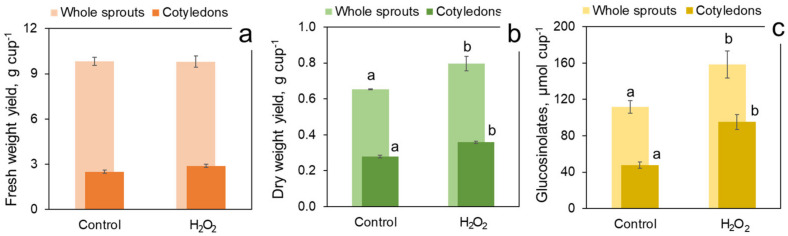
Contribution of cotyledons in fresh (**a**) and dry (**b**) yield and in glucosinolate content (**c**) of 7-day broccoli sprouts treated with 1000 mM H_2_O_2_ vs. water-sprayed control. All values are calculated per growth container. The sprouts were grown at 22 °C under illumination regime specified in Figure 5. Error bars represent standard errors of three independent biological replications. Different letters above bars indicate significant difference between the control and treatment values according to post-hoc Tukey’s HSD test (*p* ≤ 0.05). Absence of letters means that the values were not significantly different according to ANOVA test.

The trial presented in Figure 8 investigated the role of cotyledons in the sprout response to H_2_O_2_ under regular photoperiod conditions. Cotyledons represented 25–30% of the sprout fresh weight but more than 40% of the dry weight. H_2_O_2_ exposure had no effect on either total fresh yield of sprouts or on the weight of fresh cotyledons per cup (Figure 8a). At the same time, the treatment resulted in a significant increase in the yield of dry sprouts and dry cotyledons weight (Figure 8b), indicating that H_2_O_2_ treatment made the sprouts and primarily the cotyledons denser in dry matter. In Figure 8c, it could be seen that H_2_O_2_ treatment significantly increased the content of glucosinolates by approximately 47 µmol per cup, both in whole sprouts (158.7 vs. 111.7 µmol cup^−1^ in the treated and in the control sprouts, resp.) and in the cotyledons (95.4 vs. 47.8 µmol cup^−1^). Therefore, the added glucosinolate amount was entirely contributed by cotyledons, while the treatment had no effect on the glucosinolate content in other sprout parts (hypocotyls and roots), calculated as the difference between the whole sprout and the cotyledon glucosinolate contents.

### 3.3. Effect of 1000 mM H_2_O_2_ Treatment on Wild Rocket Leaves

**Figure 9:**
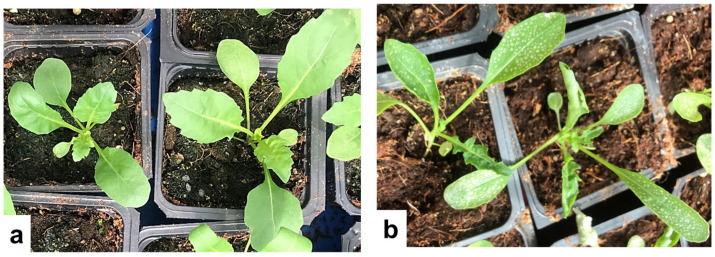
The appearance of 24-day greenhouse-grown wild rocket plants sprayed for 4 consecutive days starting from day 19 with 1000 mM H_2_O_2_ (**b**) in comparison with water-sprayed control (**a**).

In addition to the experiments performed with broccoli sprouts, a trial was performed in order to evaluate if the H_2_O_2_ treatment could bring a similar outcome with leafy vegetable from the same Brassicaceae family. The preharvest 1000 mM H_2_O_2_ treatment was applied in the greenhouse to wild rocket, or arugula (*Diplotaxis tenuifolia*), according to the same 5-day spraying protocol as with broccoli sprouts, with water spraying in the control. The treatment had a phytotoxic effect on the rocket leaves manifested as numerous bleached spots and leaves’ rolling (Figure 9). Even though a marked difference on the leaves’ appearance was observed, there was no significant yield difference between both groups.

The effect of H_2_O_2_ treatment on the phytonutrients content in rocket leaves followed a pattern similar to that with broccoli sprouts. The H_2_O_2_ spray tended to increase the glucosinolate contents in rocket leaves by 26% and 33% on a fresh and dry basis, respectively. However, in contrast to the sprouts, this result was not statistically significant. On the other hand, there was a significant 25% decrease in the phenolics content in fresh H_2_O_2_-treated leaves as compared to the controls (data not shown).

### 3.4. Effect of H_2_O_2_ Treatment of Broccoli Sprouts on the Expression of Genes Related to Glucosinolate Production

Considering the significant increase in the glucosinolate content in broccoli sprouts treated with 1000 mM H_2_O_2_, a preliminary study was conducted for elucidating the molecular mechanisms of this phenomenon. The selection of genes for this study was based on their importance for the glucosinolate pathway, previous data on their expression under abiotic stress conditions and in particular the involvement of the redox status in their regulation. The genes picked for the analysis included two transcription factors influencing the biosynthesis paths of aliphatic (MYB29) and indole (MYB51) glucosinolates [19,44]. CYP (Cytochrome P450) genes were selected as genes involved in the core biosynthesis of aliphatic (CYP79F1) and indole (CYP79B3) glucosinolates. Additionally, GSH1 (γ-glutamylcysteine synthetase) and APK1 (APS kinase) were the other two genes selected because they are involved in redox regulation for glucosinolates synthesis.

**Figure 10:**
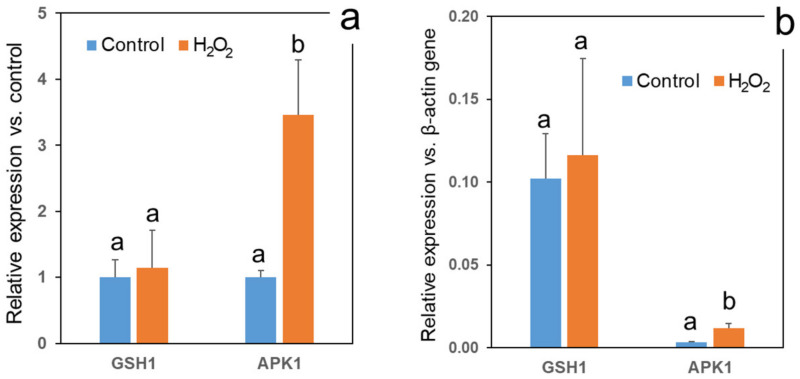
The effect of a single H_2_O_2_ (1000 mM) application on day 2 on relative expression of genes involved in sulfur mobilization for glucosinolate biosynthesis (APK1) and glutathione biosynthesis (GSH1) in 3-day, dark-grown broccoli sprouts as compared with water-sprayed control. The expression values normalized against the expression of the same gene in water-treated control (**a**) and against the expression of a housekeeping β-actin gene (**b**). Error bars represent standard errors of three biological independent replications. Bars marked by different letters indicate significantly difference between H_2_O_2_ treatment and water control values according to *t*-test comparison (*p* ≤ 0.05).

Figure 10 focuses on the expression of two competing broccoli genes involved in the mobilization of sulfur either for primary metabolism (GSH1), i.e., production of amino acid cysteine and antioxidant glutathione, or for secondary metabolism, i.e., glucosinolate biosynthesis (APK1). Figure 10a compares the expression of these genes in the H_2_O_2_-treated vs. the control sprouts, revealing a signigicant 3.5-fold upregulation of APK1 by H_2_O_2_ concomitant with glucosinolate elicitation. The expression of GSH1 was practically unaffected by H_2_O_2_. On the other hand, Figure 10b presents the expression of the two genes on a comparable basis, relative to a housekeeping β-actin gene. According to Figure 10b, sulfur was predominantly used in the sprouts for primary metabolism, in spite of the significant stimulation of the glucosinolates formation by H_2_O_2_.

## 4. Discussion

### 4.1. Eliciting Effect of Hydrogen Peroxide (H_2_O_2_)

Undoubtedly, the high eliciting potential of hydrogen peroxide (500–1000 mM) stimulating the glucosinolate biosynthesis in broccoli sprouts is the major finding of this study. To the best of our knowledge, no previous reports on H_2_O_2_ as an elicitor of glucosinolate production in *Brassicaceae* sprouts have been published previously, and relevant information for adult plant organs is very scarce [23,36]. Doubling the glucosinolate content in response to H_2_O_2_ is one of the highest upsurges of glucosinolate content in broccoli sprouts reported. Most studies by now reached 20 to 30% addition in glucosinolate content by various elicitors, e.g., methyl jasmonate (MeJa), salicylic acid, methionine, chitosan and UV-B [45,46], and only rarely reported an increase of 80 to 98% [40,47,48]. An even higher increase was achieved by combining MeJa with UV-A or UV-B [40] or by sugar feeding [49].

Plants, due to their aerobic metabolism, normally produce reactive oxygen species (ROS) such as H_2_O_2_ at basal levels. Hydrogen peroxide is a versatile molecule involved in plant stress signaling and the regulation of developmental processes and at the same time in direct antimicrobial protection [33,34]. However, excessive H_2_O_2_ concentrations can cause cell death [50,51], and therefore, its level is under the control of the antioxidative system [31]. Several authors observed the enhanced accumulation of phenolic antioxidant compounds in response to H_2_O_2_ in edible sprouts, in particular of the *Fabaceae* family. Studies on lentil sprouts revealed no differences in biomass after H_2_O_2_ treatments but an increase in some phenolic compounds [52]. The application of 20 to 30 mM H_2_O_2_ on lentil and chickpea sprouts showed the best results in total phenolics and antioxidant activity [30,53] while in mung bean sprouts, the levels of phenolic compounds increased after the exogenous application of 400 mM of H_2_O_2_ [29]. Conversely, in our studies with broccoli sprouts and rocket leaves, the phenolics content remained unchanged or slightly decreased after the application of H_2_O_2_. The reduced content of phenolic compounds and antioxidant activity was observed in H_2_O_2_-treated, fresh-cut tomatoes [54]. It cannot be excluded that the decline of phenolic antioxidant compounds observed in our trials might be due to their depletion and chemical or enzymatic degradation in the course of H_2_O_2_ scavenging [55,56,57].

### 4.2. Responses of Sprout and Plant Organs to High H_2_O_2_ Stress

Broccoli sprouts subjected in our study to high H_2_O_2_ concentrations revealed distinct phenotypical characteristics indicating their stressed state: inhibited elongation of hypocotyls and especially of roots, altered root morphology and their negative tropism and growing upwards seemingly in an attempt to escape the contact with hydrogen peroxide solution drained to the bottom of the double-cup container. It has been reported that different abiotic stresses can inhibit root elongation [58]. In particular, the application of H_2_O_2_ to *Arabidopsis* affected root growth and development due to oxidative stress [59]. At the same time, neither fresh nor dry sprout yield declined in our trials. Presumably, shortening of the sprouts was compensated by the increase in their thickness, density and/or number per cup.

It should be noted that in our trials, similar to the typical industrial practice [37], the seeds were germinated on water so that their stored nutrients (and later, also photosynthesis products) were the only energy and material sources for sprout growth and metabolism. Such a situation may create competition between the secondary metabolism enhanced by elicitation and normal sprout development, resulting in sprout distortion, even though no yield decline was registered in the H_2_O_2_-treated sprouts. We have found recently that combined H_2_O_2_ and oligosaccharide application partially alleviated the H_2_O_2_-caused morphological anomalities of sprouts and significantly stimulated their yield and phenolic and glucosinolate contents [60]. On the other hand, without H_2_O_2_ elicitation, fertilization of broccoli sprouts with inorganic [61] or organic (amino acid) [62] sulfur and nitrogen sources had only insignificant effect on glucosinolate production.

Cotyledons were revealed in our study as the site of enhanced glucosinolate accumulation in response to H_2_O_2_ treatment. In the same way, Pérez-Balibrea et al. [63] described the broccoli cotyledons as the organ with the highest bioactive compounds accumulation (vitamin C, glucosinolates and phenolics). The cotyledons showed no bleaching in response to H_2_O_2_ application on their surface, in contrast to real leaves of the rocket plant subjected to the same treatment. The better ability of sprouts, as compared to adult plant organs, to mitigate the H_2_O_2_ phytotoxicity might be related to their stronger enzymatic and non-enzymatic antioxidant protection [64]. Similarly, the *Arabidopsis* seedlings had higher antioxidant capacity than mature leaves, defending them from energy imbalance in the mitochondria [65]. Notably, the same H_2_O_2_ treatment caused stronger glucosinolate stimulation in sprouts than in the rocket leaves. The richness of broccoli sprouts in glucosinolates far exceeding their content in mature plant organs is well known [15].

In the trials with greenhouse-grown wild rocket, a leaf toxicity of 1000 mM H_2_O_2_ was evident as scattered bleaching of the leaves within 24 h after application. This result was similar to the findings of Eicher-Sodo et al. [66], who observed damage on arugula leaves after daily foliar H_2_O_2_ treatment for 11 days using milder H_2_O_2_ concentrations compared with our study. H_2_O_2_ leads to programmed cell death as a defense mechanism of the plants [67]. On the other hand, optimizing the treatment conditions might alleviate its negative effects and maximize the positive ones. For example, foliar treatment of the greenhouse-grown mustard plants with 200 mM H_2_O_2_ resulted in greater glucosinolate stimulation than the higher dose of 400 mM [36]. While the modest 17% increase in glucosinolate content in mustard leaves was similar to our results with the leaves of wild rocket, the flowers and seeds of H_2_O_2_-treated mustard plant showed much more significant increases of 96% and 78%, respectively. On the contrary, the H_2_O_2_ treatment significantly reduced the glucosinolate content in mustard roots [36]. The relationship between peculiarities of enzymatic and non-enzymatic antioxidant systems in different plant species and organs and their response to ROS elicitors such as H_2_O_2_ deserves further investigation.

### 4.3. Effect of H_2_O_2_ Treatment on Genes Expression

Considering the H_2_O_2_-induced upsurge in the total glucosinolates level, we hypothesized that this phenomenon could be associated with the upregulation of the relevant genes’ expression. The preliminary study included two transcription factor genes regulating the pathways of aliphatic (MYB29) and indole (MYB51) glucosinolates, a gene responsible for involving tryptophan in the indole glucosinolates biosynthesis (CYP79B3), and two redox-regulated genes dealing with sulfur mobilization into competing pathways to glucosinolates (APK1) or to the antioxidant glutathione (GSH1). Although the H_2_O_2_-treated sprouts showed a somewhat higher expression of all genes listed, a statistically significant relative expression difference between the treatment and the control was found only for APK1. This upregulation was coherent with the activation of the glucosinolate synthesis by H_2_O_2_ because APK1 and APK2 are important for glucosinolates formation [68]. The lack of significance of the other genes tested does not necessarily mean that they were not affected by H_2_O_2_. The detection of such effects might need a more detailed time course study of gene expression.

APK is involved in providing sulfur for glucosinolates formation in a form of adenosine 5′–phosphosulfate (APS). The APS can undergo two alternative metabolic directions: either reduction by APS reductase to form cysteine and further gluthathione by GSH1 [65], or phosphorylation by APS kinase (APK, in particular APK1) to form 3′-phosphoadenosine 5′-phosphosulfate (PAPS), which donates the sulfur group to glucosinolates formation [69]. Thus, sulfur groups can be partitioned into the primary metabolism, e.g., cysteine and glutathione, and secondary metabolism, e.g., glucosinolates [70]. It is accepted that the balance between APS reductase and APS kinase is redox-regulated. APS reductase is upregulated at an oxidative state, neutralizing the oxidative stress by enhanced glutathione activity, while APS kinase activity prevails at a reduced state [26]. Nevertheless, our results apparently contradict this scheme because a significant upregulation of APK1 was caused by an oxidizing factor, H_2_O_2_. This phenomenon may be associated with a constitutive high antioxidant status of broccoli sprouts rich in enzymatic and non-enzymatic antioxidants [71,72]. Indeed, when presented in comparable form, relative to the expression of a housekeeping gene β-actin, the expression of GSH1 gene in our analyses was much higher than that of APK1, irrespective of H_2_O_2_ treatment. Further study is needed to check if the redox homeostasis of broccoli sprouts after H_2_O_2_ treatment can justify the APK1 upregulation leading to enhanced glucosinolates formation.

## 5. Conclusions

The research has shown that H_2_O_2_ in high concentrations (500–1000 mM) is a prominent elicitor for the enrichment of broccoli sprouts with glucosinolates without a negative effect on their yield. However, an attempt to extend this approach to wild rocket plants in the greenhouse resulted in phytotoxic damage to the leaves and limited glucosinolates enhancement. The study has made the initial steps towards understanding the mechanisms of the H_2_O_2_ effect by investigating the relevant genes expression. The first encouraging finding was the effects of H_2_O_2_ on upregulating the glucosinolate-associated gene APK1. Further methodological improvements are needed in order to decipher the elicitation fenomenon and to develop a capability to manipulate it in desirable direction.

## Data Availability

The data presented in this study are available on request from the corresponding author.
